# The Effect of Wearing a Mask on Facial Attractiveness

**DOI:** 10.1093/asjof/ojac070

**Published:** 2022-09-02

**Authors:** Brian Bassiri-Tehrani, Alvin Nguyen, Akriti Choudhary, Jiddu Guart, Bianca Di Chiaro, Chad A Purnell

**Affiliations:** Aesthetic plastic surgery fellow, The Center for Plastic Surgery at MetroDerm/Emory Aesthetic Center, Atlanta, GA, USA; Medical student; Research fellow; Postgraduate year 2 resident, Division of General Surgery, Brown University, Providence, RI, USA; Postgraduate year 3 resident, Division of Plastic & Reconstructive Surgery, Loyola University Medical Center, Chicago, IL, USA; Assistant professor, Division of Plastic, Reconstructive & Cosmetic Surgery University of Illinois College of Medicine, Chicago, IL, USA

## Abstract

**Background:**

The COVID-19 pandemic necessitated masking in public spaces. Masks may impact the perceived attractiveness of individuals and hence, interpersonal relations.

**Objectives:**

To determine if facial coverings affect attractiveness.

**Methods:**

An online survey was conducted using 114 headshot images, 2 each—unmasked and masked—of 57 individuals. Two hundred and seven participants rated them on an ordinal scale from 1 (least attractive) to 10 (most attractive). Parametric and nonparametric tests were performed, as appropriate, for comparison.

**Results:**

For the first quartile, the average rating increased significantly when wearing a mask (5.89 ± 0.29 and 6.54 ± 0.67; *P* = 0.01). For control images ranked within the fourth quartile, the average rating decreased significantly when wearing a mask (7.60 ± 0.26 and 6.62 ± 0.55; *P* < 0.001). In the female subgroup (n = 34), there was a small increase in average rating when masked, whereas in the male subgroup (n = 23), there was a small decrease in average rating when masked, but the change was not statistically significant (*P* > 0.05). For unmasked female images ranked within the first quartile, the average rating increased significantly when wearing a mask (5.77 ± 0.27 and 6.76 ± 0.36; *P* = 0.001). For the female subgroup with mean ratings within the fourth quartile, the average decreased significantly when wearing a medical mask (7.53 ± 0.30 and 6.77 ± 0.53; *P* < 0.05). For unmasked male images ranked within the first quartile, the average rating increased when wearing a medical mask but the change was not statistically significant (*P* > 0.05), whereas for the control male images within the fourth quartile, the average rating decreased significantly when masked (7.72 ± 0.18 and 6.50 ± 0.54; *P* < 0.05).

**Conclusions:**

While wearing a facial covering significantly increased attractiveness for images less attractive at baseline, and decreased attractiveness for those that are more attractive at baseline; it did not cause a significant overall change in attractiveness in the study population.

**Level of Evidence: 5:**

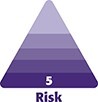

Physical attractiveness has social consequences; thus, anything that can potentially interfere with or augment facial beauty is important to interpersonal relationships. It has been well described that people who are perceived as good-looking benefit from a beauty bias or “halo” effect.^1^ They are more successful in dating aspirations, making new friends, ascending professional careers, and being exonerated for crimes compared with their less attractive counterparts.^1^ In a study, Maestripieri et al. offers an evolutionary explanation, stating it is our primal instinct to behave positively toward attractive individuals out of an overgeneralized romantic feeling toward them.^2^ While this may be debatable, there is a clear link between overall success and physical attractiveness. In fact, a person’s first impression can go a long way in changing the trajectory of their life.^3^ Several studies have corroborated that in a mere fraction of a second, opinions can be formed simply by a facial expression.^4-8^ Certain facial features can lead to judgments on personality traits (ie, likeability, trustworthiness, competence), for instance, large eyes have been shown to make people appear more empathetic, agreeable, extroverted, conscientious, and intelligent.^9^

Facial attractiveness is also instrumental in sexual selection—women have been reported to prefer deep-set eyes and large jaws in men while a smaller nose and a more symmetrical face are preferred in female faces.^10,11^ A number of factors have been reported to be predictive of facial attractiveness, namely, symmetry, averageness, sexual dimorphism, environment, exposure, skin health and color, age, adiposity, hair and eye color, facial hair in men, and make-up use in women.^1,12^ Although there is cross-cultural and gender-specific variability in what is traditionally considered beautiful, there is something universal about attractive faces.^10,13^ Galton reported that average faces, that is, multiple faces blended together were more attractive than the constituent faces.^14^ It is the harmony of the facial features, irrespective of gender, race, and ethnicity, that is considered the most instrumental in deciding the first impression of attractiveness.^12,15^ It is thus implicit that the obstruction of any zone of the face could effectively skew the perception of this attractiveness, by shifting the importance to unoccluded features disproportionately.

The spread of COVID-19, which was declared a global pandemic by the WHO on March 11, 2020,^16^ has changed the world in significant ways.^17^ Specifically, masking protocols put in place to curtail the risk of COVID-19 transmission^18,19^ have affected the psychosocial interactions among people.^4-8^ In this era of widespread masking, individuals with blemishes or deformities affecting their mid and lower face may find that wearing a mask hides most or all of their imperfections, leading to improved perceived facial aesthetic due to occlusion. Conversely, it may disadvantage those with upper facial and peri-orbital flaws, as masks may accentuate them. This may have pertinent implications both on individuals and society as a whole.

The pre-pandemic effect of partial occlusion of the lower half of the face on the perceived attractiveness of female faces was demonstrated in the Japanese population by Miyakazi et al in 2016.^20^ They demonstrated an “occlusion effect” vs a “sanitary mask effect,” specific to medical masks. In this study, medical masks in particular resulted in decreased attractiveness compared to occlusion with other objects, presumably due to the association of illness with wearing a mask. Kamatani et al, in 2021, demonstrated the role of the global pandemic in eliminating the “sanitary mask effect” due to the ubiquity of masks.^21^ Similarly, other studies conducted in Canada,^22,23^ UK,^24^ and Spanish-speaking countries^25^ have attempted to understand the association of partial facial occlusion (by masking, control objects and image manipulation) with perceived attractiveness, and its interaction with base attractiveness of nonoccluded faces. Patel et al^26^ conducted a larger survey in 2020 at a time when the global population was starting to acclimate to the mandated widespread practice of wearing facial coverings.

After having experienced multiple surges of the COVID-19 pandemic, facial masking is now a commonplace practice in public spaces and a routine part of one’s daily life. We conducted a crowdsourcing-based cross-sectional study in October 2021, to assess the perception of masked facial attractiveness at this stage of the pandemic, when one sees a large proportion of strangers using face masks and is relatively comfortable with the practice. The primary aim of our study is to evaluate to what extent, if any, wearing a mask affects facial attractiveness. Our secondary aim is to determine if cohorts that are perceived the least or most attractive at baseline were differently affected by wearing a mask.

For crowdsourcing, we utilized Amazon Mechanical Turk (MTurk, [Amazon Web Services, Amazon, Seattle, WA]), which has been used in the past as an effective platform for online crowdsourcing in scientific and nonscientific applications.^27-29^ It provides a more economical, faster, and anonymous method of recruiting participants as compared to prospective longitudinal enrolment.^30^ Moreover, it offers a population self-reported as being representative of the US population with respect to gender, race, age, and educational qualifications.^27,30-32^ In the field of plastic surgery, MTurk has been previously utilized to assess public perception of aesthetic features.^33-36^

## METHODS

We designed a cross-sectional survey to compare perceived attractiveness between unmasked and masked images of the same individuals. An a priori power analysis determined that a minimum of 100 total photographs would be needed (50 unmasked and 50 masked) in order to determine a 2-point change on a 10-point scale, with a power of 0.8 and significance set to 0.05.

**Figure 1.**
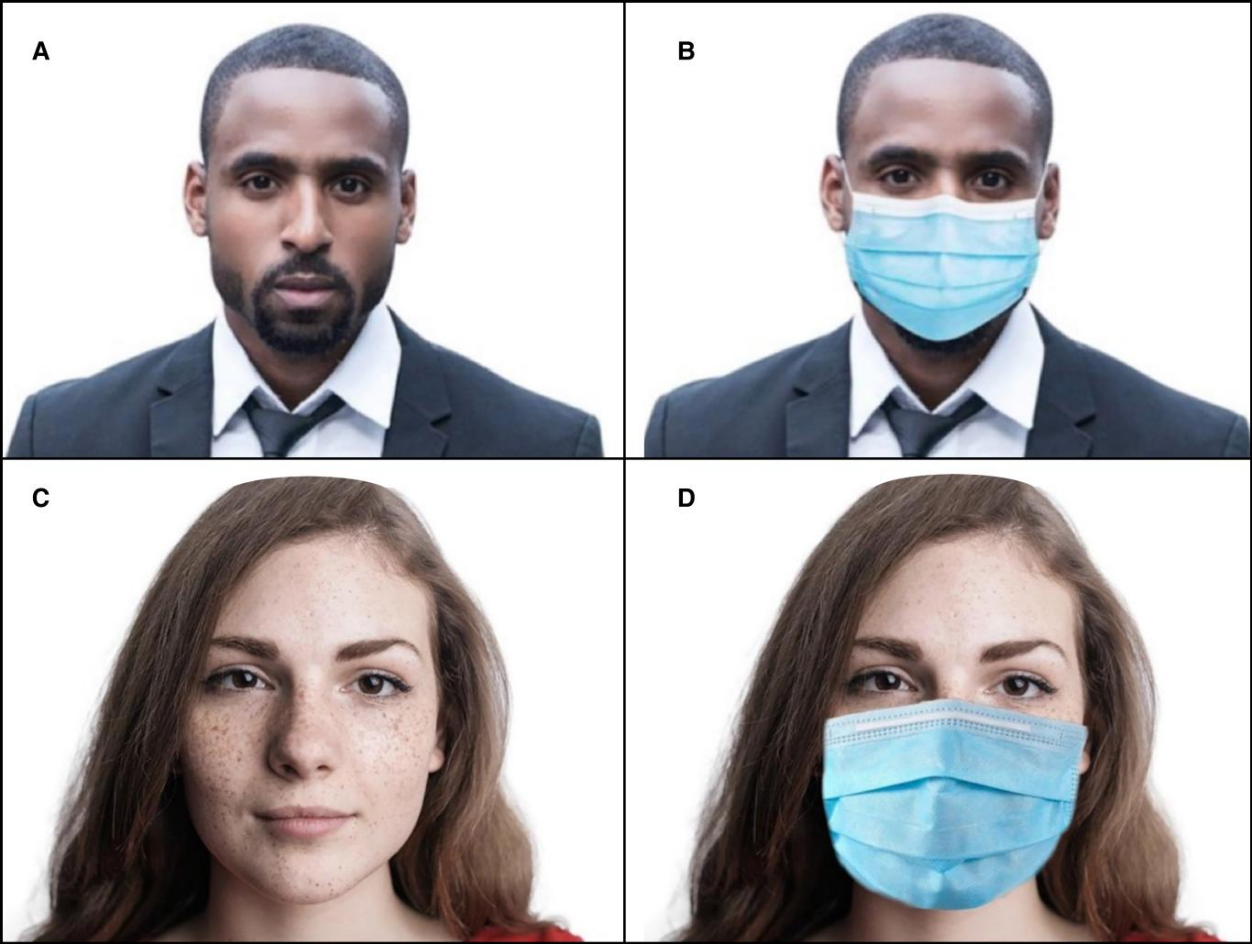
Example of images used in the (A and C) control and (B and D) experimental groups. Image courtesy of Pexels stock photography (https://www.pexels.com).

Anteroposterior headshot images of 57 subjects (34 female, 23 male) with neutral facial expressions were collected from Pexels (Fuldabrück, Hessen, Germany), an online public-domain provider of free-stock photos. Images with visible tattoos and facial markings (scars and nevi) were excluded. No restriction was placed on race, ethnicity, age, hairstyle, skin texture, and eyewear use. Using Adobe Photoshop (Adobe Inc., San Jose, CA), the images were put into standardized form with a plain white background. The control group consisted of these facial images without any facial coverings. These images were then electronically modified within Photoshop to simulate a blue medical mask as a uniform face covering, serving as the experimental group. Unnatural mask edges were blurred using features within Adobe Photoshop (Figure 1).

Utilizing Research Electronic Data Capture ([REDCap] Nashville, TN), an online survey was created consisting of images of both the groups mixed together in a randomized order. Randomization was performed by numbering the images and using an online random sequence generator. Survey participants were recruited using Mturk a crowdsourcing marketplace. To qualify for participation in the survey, the MTurk workers were required to have an HIT (Human Intelligence Task) Approval Rate of 85% or more. The identity of these participants was unknown. Details or aims of the study were not disclosed in the disclaimer of the survey. The participants received instructions to rate the images on an ordinal scale ranging from 1 (least attractive) to 10 (most attractive). Participants were given an option to retract their responses at any point during the survey. Every participant was paid $1.50US upon completion of the survey. Partially completed surveys were excluded.

The subjects were stratified based on gender (male and female) and average ratings (first and fourth quartiles). Descriptive statistics were used to summarize the outcome measures (means, medians, standard deviations) and the measures of central tendency were compared using appropriate statistical tests on SPSS (Statistical Package for the Social Sciences, IBM SPSS Version 28.0.1.0, IBM, Armonk, NY). Statistical significance was defined as *P* < 0.05.

## RESULTS

Our survey included anteroposterior facial photographs of 34 female and 23 male, racially diverse subjects. A total of 207 MTurk participants completed the survey in October 2021. We included 114 photographs (57 unmasked and 57 with masks) in our study; this yielded 23,598 data points in total. The mean attractiveness score was 6.74 (±0.67) for unmasked subjects and 6.70 (±0.61) for masked subjects (*P* = 0.75) (Table 1).

**Table 1.** Mean Ratings of Groups and Gender-Based Subgroups

Gender	Control (no mask) mean (SD)	Experiment (masked) mean (SD)	*P*-value^a^
Overall	6.74 (0.67)	6.70 (0.61)	0.75
Female (n = 34)	6.69 (0.69)	6.80 (0.51)	0.49
Male (n = 23)	6.80 (0.66)	6.55 (0.72)	0.20

SD, standard deviation.

a *P* < 0.05—statistically significant, *P* < 0.001—highly statistically significant.

**Figure 2.**
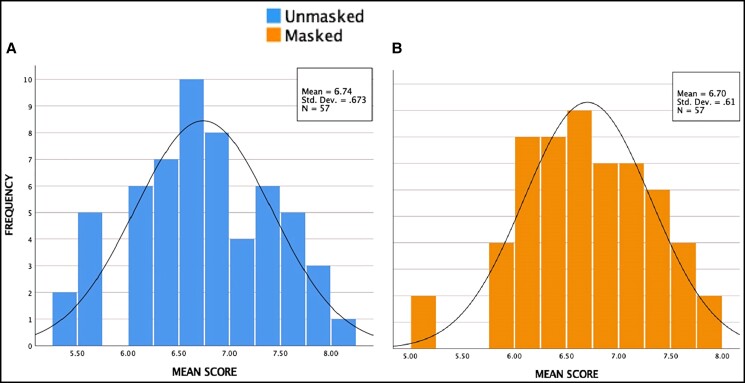
Frequency distribution of mean ratings of the (A) control group and (B) experimental group. *P* < 0.05 is statistically significant.

On obtaining the average ratings for each of the 114 images, the distribution of means yielded a bell-shaped normal curve, both at baseline and when masked (Figure 2). The mean ratings for the control group and the experimental group images ranged from 5.48 to 8.10 and 5.13 to 7.92, respectively.

### Subgroup analyses

**Figure 3.**
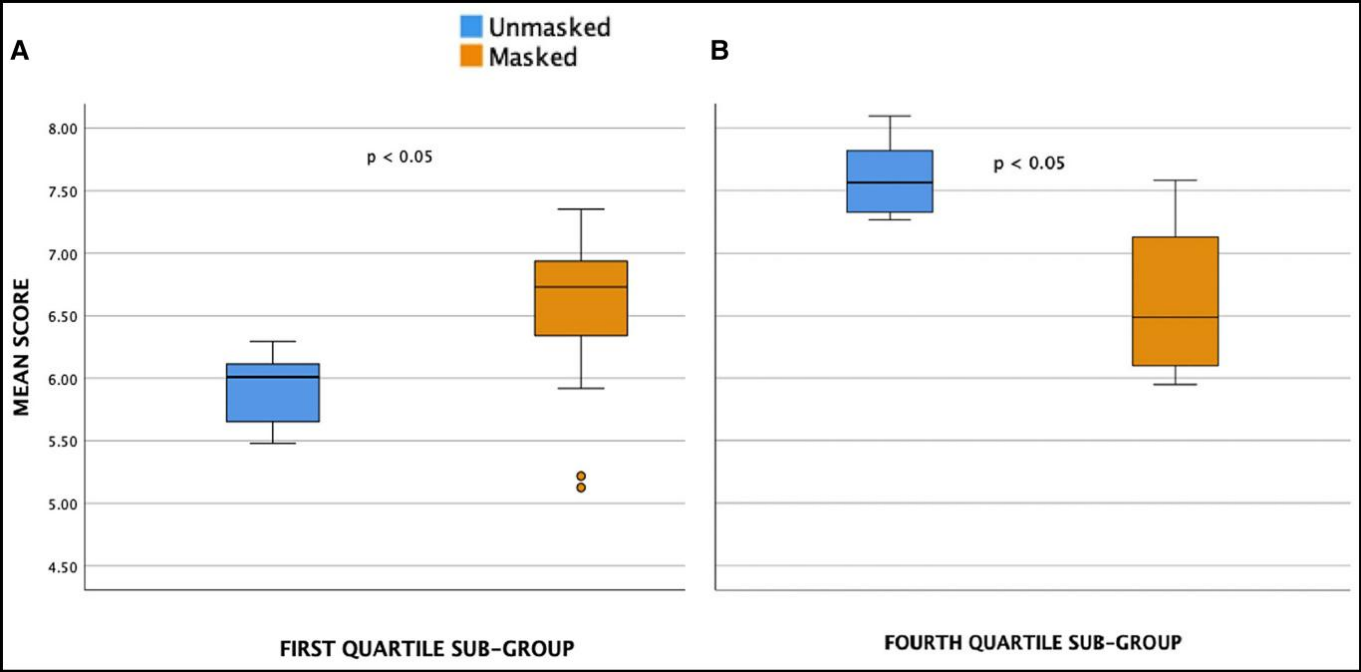
Box plot demonstrating change in the mean ratings for overall: (A) first quartile and (B) fourth quartile. *P* < 0.05 is statistically significant.

Of the 57 control images, 15 were in the first quartile of distribution (lowest rated) while 14 were in the fourth quartile of distribution (highest rated). For the first quartile, the average rating increased significantly when wearing a mask (5.89 ± 0.29 and 6.54 ± 0.67; *P* = 0.01). For control images ranked within the fourth quartile, the average rating decreased significantly when wearing a mask (7.60 ± 0.26 and 6.62 ± 0.55; *P* < 0.001) (Figure 3). In the female subgroup (n = 34), there was a small increase in average rating when masked, whereas in the male subgroup (n = 23), there was a small decrease in average rating when masked, but the change was not statistically significant (Table 1).

**Figure 4.**
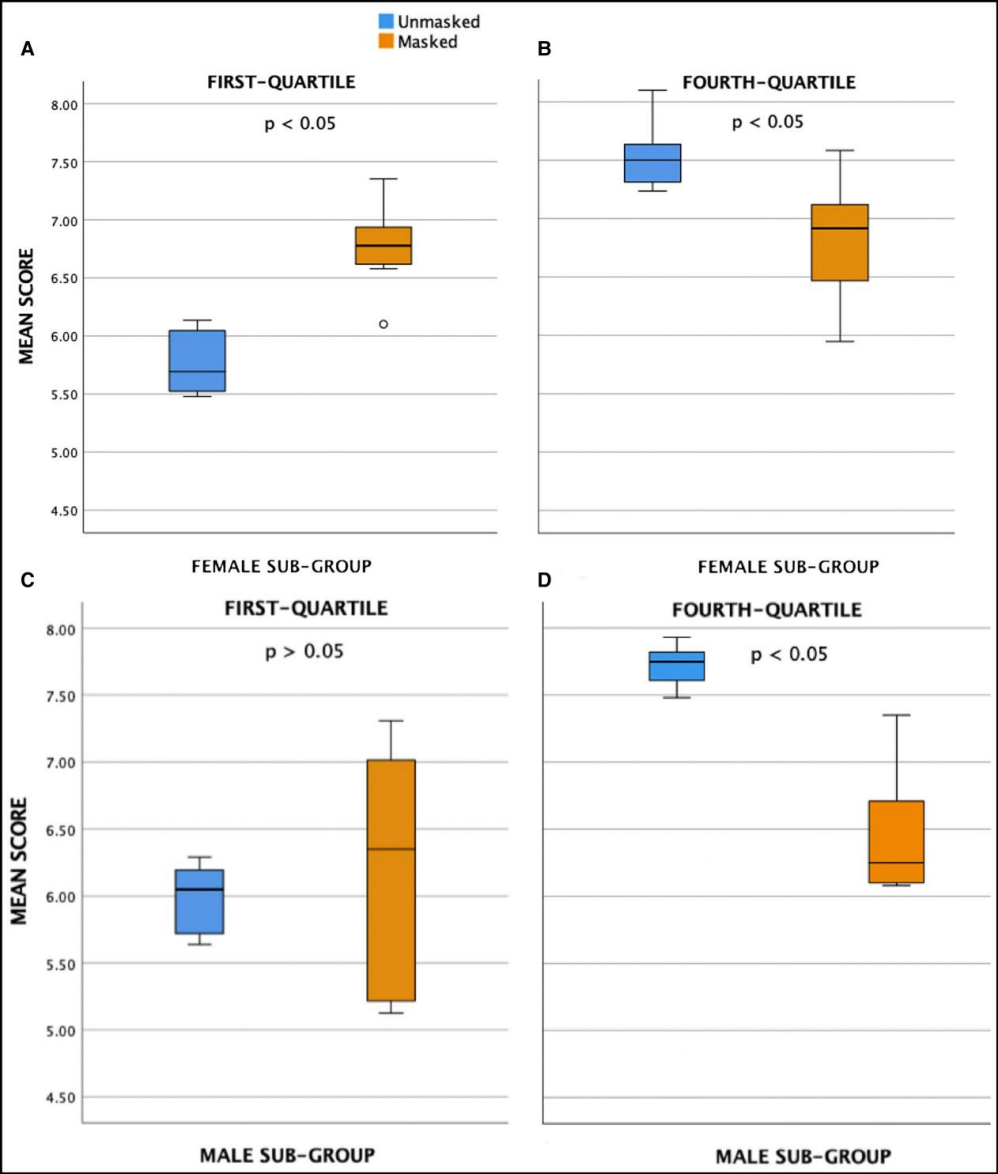
Box plot demonstrating change in the mean ratings for gender-stratified subgroups: (A) female first quartile; (B) female fourth quartile; (C) male first quartile; and (D) male fourth quartile. *P* < 0.05 is statistically significant.

For unmasked female images ranked within the first quartile, the average rating increased significantly when wearing a mask (5.77 ± 0.27 and 6.76 ± 0.36; *P* = 0.001). For the female subgroup with mean ratings within the fourth quartile, the average decreased significantly when wearing a medical mask (7.53 ± 0.30 and 6.77 ± 0.53; *P* < 0.05). There was decreased variability with masking by quartile in male subjects. For unmasked male images ranked within the first quartile, the average rating increased when wearing a medical mask but the change was not statistically significant (*P* > 0.05), whereas for the control male images within the fourth quartile, the average rating decreased significantly when masked (7.72 ± 0.18 and 6.50 ± 0.54; *p* < 0.05) (Figure 4).

## DISCUSSION

Routine mask-wearing has become a part of life worldwide during the COVID-19 pandemic. While reduction of viral transmission is the primary goal of masking, it has many unintended consequences—it can interfere with communication, especially among the hearing impaired.^26^ Masking can also affect exercise tolerance and change how social cues are interpreted.^37^ Although not its most critical effect, there is evidence that wearing facial covering results in changes to perceived attractiveness.^20,21,24-26^

“The eyes are the window to the soul”: indeed, peri-orbital aesthetics play a crucial role in determining one’s attractiveness.^38^ Similarly, the nose is a significant determinant of facial harmony and overall facial aesthetics.^39^ With regard to oral and peri-oral aesthetics, while full lips with the presence of well-defined philtral contours are equated to youthfulness and beauty,^40,41^ a long, ill-defined philtrum with a thin upper lip is linked to aging.^41^ Additionally, the absence of teeth, staining of teeth, and a crooked smile due to congenital or acquired factors are linked to lower perceived attractiveness.^41^ In the past, various studies have reported the significance of the lower third of the face and the associated sexual dimorphism in overall perceived facial attractiveness.^41-43^ Wearing masks effectively hides nasal and peri-oral asymmetry, deformity, or disproportion while simultaneously accentuating the peri-orbital region. If a subject possesses traditionally favorable peri-orbital features, wearing a mask will highlight these. Conversely, if a subject shows signs of aging, asymmetry, or deformity around the peri-orbital region, this will be more pronounced when wearing a mask.

In a previous study involving university students in Canada, Sadr and Krowicki reported that less facial information leads to more perceived attractiveness.^22^ They conducted a student survey using methods of blurring, reducing image contrast, and partial occlusion of different facial features to manipulate images. They concluded that the occlusion of facial features was positively associated with perceived attractiveness, regardless of the feature occluded and baseline perceived attractiveness for unmasked faces. Orghian et al conducted a similar study using photographs of incomplete faces in 2020 and corroborated this.^44^ Several studies also evaluated the effect of occlusion of the lower half of the face, specifically, on perceived opinions of the participant’s attractiveness and social desirability by onlookers—Miyakazi et al studied this effect using medical masks and control occluders; they concluded that control occluders, like cards and books, covering the lower half of the face worked as an equalizer in ratings for individuals of low baseline and high baseline attractiveness.^20^ There was a regression to the mean in ratings for the two groups due to an effect defined as the “occlusion effect” by the researchers. They reported that this effect did not extend to medical masks and observed lower perceived attractiveness in the masked participants as compared to baseline because of a hypothesized “sanitary mask effect.” Miyakazi et al^20^ defined “the sanitary mask” as arising due to the association of medical masks with the assumed presence of disease and poor health among the Japanese population. When this study was repeated by Kamatani et al in 2021 in Japan during the COVID-19 pandemic, using black and white masks, they observed results in agreement with the occlusion effect; no sanitary mask effect was observed.^15^ The researchers hypothesized the loss of the “sanitary mask effect” to be related to the pandemic experience which decreased the association between medical masks and poor health. There have since been varying reports on the association of face coverings with perceived attractiveness, and their interaction with baseline attractiveness, type of mask worn (cloth masks, black, white, and blue medical masks), age of the observed subjects, and control occluders. Pazhoohi et al^23^ demonstrated the importance of the peri-orbital region in the evaluation of attractiveness; they reported an increase in attractiveness ratings by masking young and old subjects with average and below-average baseline attractiveness. These findings were irrespective of whether the loss in stimulus from the lower half of the face was from wearing black masks or cropping images.^23^ These observations were not replicated in ratings of images with above-average attractiveness, or on occlusion of the upper half of the face, further highlighting the role of the peri-orbital region in facial aesthetics.^23^ In the United Kingdom, Hies et al attempted to compare the effect of medical masks, cloth masks, and control occluders on attractiveness ratings for male subjects.^24^ They reported a global increase in ratings regardless of baseline attractiveness and type of occlusion; the highest mean ratings were observed in the medical mask cohort. In 2020, Patel et al demonstrated the occlusion effect, reporting a regression to the mean in attractiveness ratings for the below-average and average attractiveness cohort, and for the above-average attractiveness cohort, on using medical masks.^26^ Overall, this evidence points toward a possible change in opinion with regard to medical mask usage among the general population, the majority of which was not used to wearing masks before the pandemic—in the pre-pandemic era,^20^ medical masks were assumed to indicate poor health while post the pandemic, medical masks led to either uniformly increased perceived attractiveness^24^ or a regression to the mean as demonstrated by Patel et al^26^ and in our study. This could be due to an increase in trust on masked individuals, considering them as responsible citizens, or due to the dissociation of the stimulus of medical masks with the assumption of illness.

**Figure 5.**
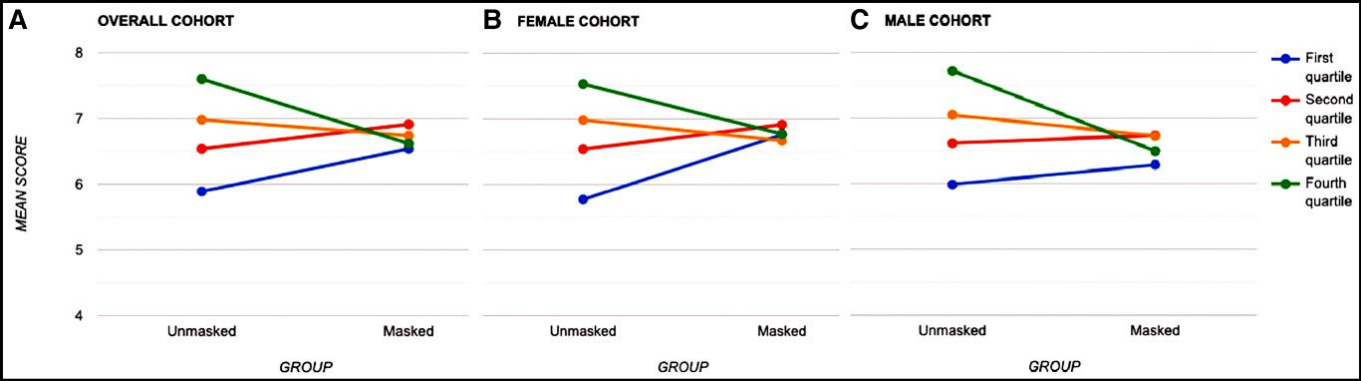
Line graph demonstrating change in ratings stratified by quartiles for the (A) overall cohort (n = 57), (B) female subgroup (n = 34), and (C) male subgroup (n = 23).

In this cross-sectional study, we attempted to assess whether the longer experience of living with the pandemic and, therefore, familiarity to the use of medical masks led to a change in the perception of the attractiveness of masked faces. Our study corroborates the previous reports demonstrating a regression to the mean in ratings for the lowest and highest rated cohorts when masked, although when evaluating the cohort as a whole, on average, wearing a mask did not significantly affect perceived attractiveness. Figure 5 illustrates the phenomenon of regression to the mean observed in the female subgroup and the overall cohort. Our observations may be hypothesized to be due to the effect that masking has on subject faces who have relatively higher attractiveness ratings at baseline, as the participants do not see the complete picture of youthfulness and facial harmony that may be present. Moreover, subjects in this cohort may have peri-orbital features that may not be deemed as favorable in relation to the rest of the face, thus leading to lower ratings. In a sense, wearing a mask “levels the playing field”; the least attractive faces improve in ratings while faces that are most attractive suffer a reduction in ratings. This finding conveys the crucial role of the lower half of the face, specifically, the facial features occluded by masks, that is, the nasal, oral, and the peri-oral regions in determining the complete facial aesthetic, in addition to the peri-orbital region. Additionally, there are gender-related differences in the relative contributions of facial regions to perceived sexual dimorphism and facial attractiveness.^45-47^ Perceived sexual dimorphism is critical as the face is a “health certificate” reflective of one’s value as a mate.^30^ Ibanez-Berganza et al reported that the facial traits significant for sexual dimorphism are the nose size, eye height, and the width of the face and the jaw.^47^ The lower third of the face has been known to play a pivotal role in the overall harmony of the female face—a squared-off jaw contributes to perceived aging, more masculinity, and less attractiveness^45^—whereas traits such as eye size and depth, eyebrow thickness, and hairline are more important to male faces.^10,11,45,47^ This is reflected in our findings of a greater effect of facial masking on the least and most attractive female faces as compared to the male subgroups; this is due to occlusion, and therefore, loss of significant stimulus from the lower portion of the female faces.

The use of facial masks effectively skews the concept of overall facial harmony and shifts the onus to the peri-orbital region and the upper third of the face. This may lead to disproportionately greater public interest in periocular cosmetic procedures; in fact, the Aesthetic Plastic Surgery National Databank Statistics 2020-21, published by The Aesthetic Society, recorded a 72% increase in the procedure count of blepharoplasty from 2020 to 2021.^48^ Blepharoplasty was also among the top 5 demanded procedures in 2021 and contributed to 6% of the total surgical revenue.^48^ Findings from this study will be interesting to correlate with aesthetic surgical statistics in the future. Given this time of increased focus on the upper face, we will perhaps see more and younger patients undergoing peri-orbital surgical and nonsurgical rejuvenation. Conversely, continued coverage of the lower face in older patients might lead to a decrease in rhytidectomy procedures in the short run.

Certain limitations in this study should be noted. Since the data points were from an online crowdsource and the sample size is large, a seemingly small change may represent statistical significance primarily due to the sample size. Thus, a statistically significant interpretation may not be clinically relevant. The changes noted among the lowest and highest quartile of attractiveness are larger and are likely more clinically relevant. Although the participants in this survey are lay people and not expert evaluators, the large sample size yielded a normal bell-shaped Gaussian curve for both the masked and unmasked groups of images. This supports the law of large numbers, which dictates that the true statistic is approached as the sample size increases. Although crowdsourcing has been used in a variety of study areas within plastic surgery^27-29,33-36,49^ and is considered an excellent method of gaining a large number of survey responses from a lay audience, there have been critiques of crowdsourcing as an approach for medical research. It specifically faces criticism for limitations in its generalizability^50^ and the probability of false results due to cognitive fixation.^51^ The large sample size utilized in our study and the use of an online de-identified platform largely addresses these concerns. One limitation of crowdsourcing of images for the evaluation of perceived attractiveness may be that online platforms with free-stock images do not usually have images of people with dentofacial deformities and therefore excludes this subpopulation.

Another limitation inherent to our study methodology is the absence of attention check questions and demographic information of the subjects and observers. Although we prescreened the MTurk workers using an HIT Approval Rate of 85% as a cut-off for participation, attention check questions or demographic questions were not incorporated in the survey. Since we required every survey-taker to rate 114 images, additional questions could contribute to survey fatigue and attrition. This is why we chose not to incorporate additional granularity in this particular study. However, further study is underway to determine more clearly which aspects of the face contribute to the changes in perceived attractiveness determined in this study. With respect to the observers, there may be a possible selection bias due to the disproportionate use of online platforms by people belonging to a certain demographic (eg, younger population and higher education level). Further, there was no information regarding the race, nationality, and age of the observers, which can affect the results due to racial, cultural, and age-related differences in perceived ideals of beauty. In case of the subjects whose images were used, a possible selection bias may exist with respect to their age.

Stronger evidence could be drawn by larger cross-continental studies with images of subjects comprising a wide range of races, ethnicities, baseline attractiveness, and dentofacial proportions. A generalized conclusion may be difficult to be drawn due to cultural differences in standards of beauty and attractiveness. The researchers conjecture that it would be interesting to determine whether the evaluation of facial attractiveness and the trend in its change on masking would be different when performed by those formally trained to study facial symmetry and proportion. In this particular study, we were focused on determining general societal trends in nonexpert participants.

## CONCLUSIONS

Wearing a medical mask covering the nose and mouth does not affect perceived attractiveness in subjects who are ranked in the middle quartiles of attractiveness at baseline but may affect those ranked in the highest and lowest quartiles for attractiveness. Those in the upper quartile can expect a decrease in perceived facial attractiveness when donning a mask while those in the lowest quartile can expect an increase in perceived attractiveness with mask usage.
